# Cranberry extract initiates intrinsic apoptosis in HL-60 cells by increasing BAD activity through inhibition of AKT phosphorylation

**DOI:** 10.1186/s12906-020-2870-4

**Published:** 2020-03-06

**Authors:** Rasha A. Mansouri, Susan S. Percival

**Affiliations:** 1 Department of Biochemistry, King Abdul Aziz University, Jeddah, Saudi Arabia; 2 Food Science and Human Nutrition Department, University of Florida, Gainesville, FL 32611 USA

**Keywords:** Cranberry, HL-60, Apoptosis, Caspases, Protein kinase B (Akt), BCL-2-associated death promoter (BAD)

## Abstract

**Background:**

Cranberry has been studied as a potential anticancer agent as it is capable of inducing apoptosis within cancer cells. The aim of this study was to better define the mechanism by which cranberry triggers apoptosis in HL-60 cells.

**Methods:**

The study was carried on cranberry extracts (CB). Anti-apoptotic B-cell lymphoma-2 (BCL-2) and pro-apoptotic BCL-2-associated death promoter death (BAD) proteins in cell lysates were detected through Western blotting techniques. Equivalent protein loading was confirmed through anti-α-tubulin antibody.

**Results:**

The results showed that treatment of HL-60 cells with CB causes a significant increase in the levels of caspase-9 and caspases-3/7 and increased mitochondrial outer membrane permeability, leading to the release of cytochrome C and Smac. These apoptotic events were associated with a significant decrease in protein kinase B (AKT) phosphorylation, which caused significant increase in BAD de-phosphorylation and promoted a sequence of events that led to intrinsic apoptosis.

**Conclusion:**

The study findings have described a molecular framework for CB-initiated apoptosis in HL-60 cells and suggested a direction for future in vivo studies investigating the anticancer effect of cranberry.

## Background

Apoptosis is a complex process involving multiple cellular signaling pathways. Two core pathways have been identified in mammalian cells: the extrinsic or death receptor pathway, and the intrinsic or mitochondrial pathway [1–3]. The underlying mechanistic basis for both pathways involves the sequential activation of a group of cysteine proteases called caspases, which can cleave substrates located in different intracellular compartments during apoptosis [4–6]. Caspases involved in apoptosis are broadly categorized into initiators (caspases 2, 8, 9 and 10) and executioners/effectors (caspases 3, 6 and 7). All caspases are produced in cells as catalytically inactive proenzymes. The activation of an executioner caspase is executed by the activation of an initiator caspase. Once the executioner caspase is activated, it initiates the proteolytic degradation of a broad spectrum of cellular targets that ultimately led to cell death [2, 7].

The extrinsic apoptotic pathway is initiated by the binding of extracellular death ligands to cell surface death receptors. Therefore, it is important to regulate cell death for cancer cells to survive during metastasis. This binding creates a cascade of events that activate the initiator caspase that is caspase-8, which then cleaves and activates the executioner caspases, caspase-3 and -7 [8–11]. In contrast, initiation of the intrinsic apoptotic pathway, by events such as DNA damage, growth factor withdrawal, or loss of contact with the extracellular matrix lead to changes in the integrity of the mitochondrial membrane that result in the release of pro-apoptotic proteins such as cytochrome C and second mitochondria-derived activator of caspases (Smac). These pro-apoptotic proteins mediate the activation of the initiator caspase that is caspase-9, which triggers the committed execution phase of apoptosis [3, 12–14]. A clear and distinct separation of two pathways is possible based on the links between the extrinsic and intrinsic pathways [14].

Cranberry is effective in treating urinary tract infections in vivo and in vitro animals as it works by inhibiting the adhesion of type I and P-fimbriated uropathogens to the uroepithelium, which impair colonization and subsequent infection [15]. Naturally occurring dietary substances have a long history of use in the treatment of cancer. Cranberries are considered to be rich in bioactive compounds and it is ranked among fruits in both antioxidant quality and quantity due to the presence of flavonoids and phenolic acids [16]. In addition, the presence of flavonoids plays a significant role in cancer inhibition. The potential anticancer activity of cranberry was reported for the first time in 1996 [17]. Evidence has emerged since then to support the anticancer properties of cranberry and a variety of possible mechanisms of action have been proposed [16].

It is important to consider the mechanism of apoptosis in cancer as it helps to understand the pathogenesis of the disease and aid in the development of drugs that can target certain apoptotic proteins or pathways. Therefore, induction of apoptosis is considered among the proposed mechanism of anticancer activity. In vitro studies have shown that cranberry triggers apoptosis through the modulation of a number of key proteins in cellular signal transduction pathways [18–21]. However, the exact mechanism that underlies how cranberry regulates and induces the apoptotic process remains unclear. Therefore, the present study aims to define the mechanism by which cranberry extract (CB) triggers apoptosis in HL-60 cells. This specific cell line was used since it is a well-characterized one and the various biochemical steps occurring during apoptosis have been well documented. In addition, it is a classical cell line model that has been used extensively in apoptosis studies.

## Methods

### Cranberry extract

The CB was obtained from Ocean Spray Cranberries, Inc. (Lakeville, MA, USA). It was derived from the juice portion of the berry and contained 60–65% proanthocyanidins (PAC), 9% flavonols, 6% anthocyanidins, and 5% phenolic acids. The composition of 15% is unknown, but is likely to be sugars, organic acids, and terpenes. For all experiments, CB was dissolved in DMSO, which was used at a final concentration of < 0.1% in cell cultures.

### Cell culture

HL-60 cells (CCL-240) were obtained from the American Type Culture Collection (Manassas, VA, USA). These cells were cultured in RPMI 1640 complete medium containing 10% FBS, 2% HEPES, 0.1% gentamicin sulfate, 100 U/ml penicillin, 100 μg/ml streptomycin, and 0.25 μg/ml amphotericin B incubated at 37 °C in a humidified, 5% CO_2_ atmosphere, as recommended.

### Dose and time determination

HL-60 cells (8 × 10^5^) in 25 cm^2^ flasks were treated with two concentrations of CB (25 or 50 μg/ml), 0.1% DMSO or 10 μM etoposide (BioVision, Inc., Milpitas, CA, USA), and incubated for 24 h for dosage determination. Cell samples were assayed using the Apo-ONE® Homegenous Caspase-3/7 Assay (Promega, Madison, WI, USA) by following manufacturer’s directions. According to the obtained results, 25 μg/ml CB was used in time dependent manner for further experiments. For optimal incubation time determination, cells were treated with 25 μg/mL CB and harvested at 2, 24 and 48 h. cells were assayed following the same previous Apo-ONE® Homegenous Caspase-3/7 assay.

### Experimental treatment

HL-60 cells were treated with 25 μg/ml CB for 24 h. Control cells received either 2 or 10 μM etoposide, or 0.1% DMSO. The positive control for the protein kinase B (AKT) activity assay was 20 μM PI3K inhibitor LY294002 (Cell Signaling Technology, Danvers, MA, USA).

### ELISA determination of protein levels

Levels of caspase 8, caspase 9, cytochrome C, and Smac in lysates of HL-60 cells treated with CB were quantified using commercially available ELISA kits (Abcam, Cambridge, MA, USA). AKT and phosphorylated-AKT (p-AKT) levels were also determined by ELISA (Thermo Fisher Scientific, Waltham, MA, USA), and p-AKT values were normalized to total AKT.

### Measurement of mitochondrial transmembrane potential

HL-60 cells were treated with CB, collected after 24 h, and processed using a Mitochondrial Transmembrane Potential Apoptosis Detection Kit (Abcam). Labeled cells were observed on an EVOS XL microscope (Thermo Fisher Scientific, AMG), using a band-pass filter to detect fluorescein and rhodamine.

### Western blot analysis

Western blotting techniques were used to detect anti-apoptotic B-cell lymphoma-2 (BCL-2) and pro-apoptotic BAD proteins in cell lysates. Briefly, CB-treated HL-60 cells were washed with ice cold PBS and lysed with RIPA buffer containing protease and phosphatase inhibitors. Samples were kept on ice and vortexed occasionally for 30 min. Protein concentrations were determined using a Bio-Rad Protein Assay. Samples were electrophoresed in 12% SDS-PAGE gels, transferred to nitrocellulose membranes, blocked with 5% BSA in TBST buffer, and exposed to rabbit monoclonal antibodies at 4 °C overnight. Blots were probed with HRP-conjugated anti-rabbit IgG for 1 h and visualized using superSignal West Pico Chemiluminescent Substrate (Thermo Fisher Scientific). Antibodies to proteins BAD [No.9239], p-BAD (Ser112) [No.5284], p-BAD (Ser136) [No.4366], p-BAD (Ser155) [No.9297], BCL-2 [No.2870], p-BCL-2 (Ser70) [No.2827], and anti-rabbit IgG-HRP [No.7074] were purchased from Cell Signaling Technology. Anti-α-tubulin antibody (Sigma-Aldrich, St. Louis, MO, USA) was used to confirm equivalent protein loading. Protein bands were quantified using AlphaView software, and the data were normalized to α-tubulin.

### Statistical analysis

The data has represented means of at least three independent experiments ± SD. Statistical analysis was performed using either a t-test or one-way ANOVA, followed by the pairwise test or Bonferroni comparison to the control. The data was not used for statistical analysis, when etoposide was employed as a positive control. Data were log transformed if normality failed or unequal variability occurred. Differences were considered significant when the *P* value was < 0.05. Statistical analyses were conducted using SigmaPlot 11.0 software (Systat Software Inc., San Jose, CA, USA).

## Results

### Effect of CB on caspase-3/7 activity

**Fig. 1:**
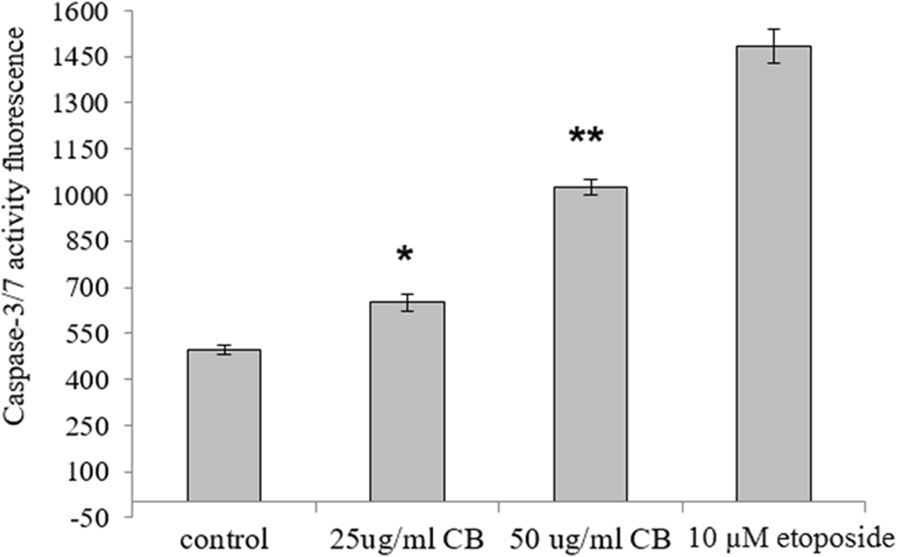
Change in caspase-3/7 activity after 24-h incubation with different concentration of CB (25 and 50 μg/mL) and etoposide (10 μM). DMSO (0.1%)-treated cells served as controls. The data represent the mean of three independent experiments±SD Statistical analysis were performed by one-way variance analysis (ANOVA) followed by Bonferroni’s test. Statistical difference shown as *P* < 0.05 [*]

**Fig. 2:**
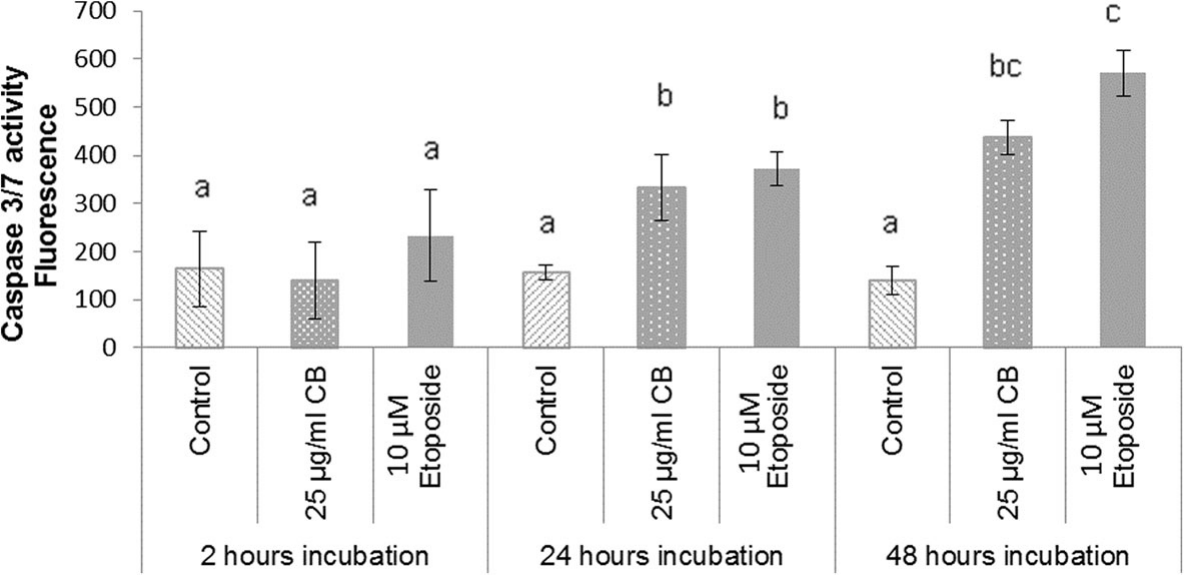
Change in caspase-3/7 activity at different incubation time under the effect of 25 μg/mL CB and 10 μM etoposide treatments. DMSO (0.1%)-treated cells served as controls. The data represent the mean of three independent experiments±SD Statistical analysis were performed by one-way variance analysis (ANOVA). Different letters indicate significant differences among the means after Bonferroni’s test (*P* < 0.05)

Initial dose and time dependent experiments were used to determine the minimal CB amount that would initiate apoptosis. Dose determination results showed that CB treatment of HL-60 cells for 24 h led to significant increases in caspase-3/7 activity at both 25 (*P* < 0.05) and 50 μg/ml (*P* < 0.001) (Fig. 1). The incubation time study showed no significant changes in caspase 3/7 activity in cells treated with 25 μg of CB for 2 h; however, significant increase in cell’s activity was observed after incubation for 24 and 48 h (*P* < 0.05). There was no significant difference between the two time points (Fig. 2). These results indicated that 24 h treatment of HL-60 cells with 25 μg/ml CB could increase the activity of caspase 3/7 and initiate an apoptosis pathway and consequently these conditions were chosen to be applied for all further experiments.

### Effect of CB on caspase-8 and -9 concentrations

**Fig. 3:**
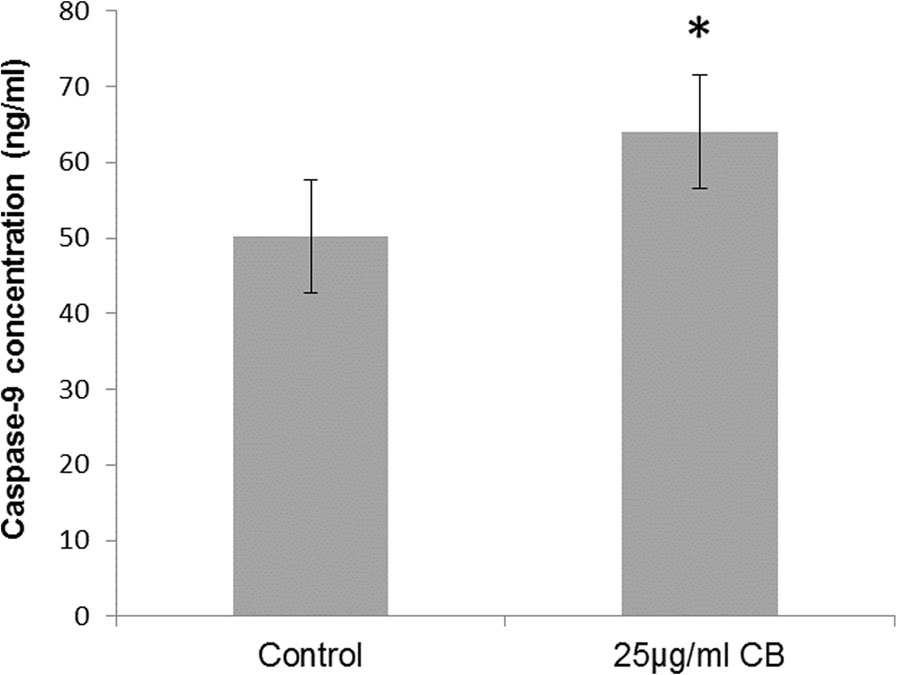
Change in caspase-9 concentration. Cells were treated with 25 μg/mL CB for 24 h. Control or DMSO (0.1%)-treated cells served as controls. Cell lysate was diluted two-fold with the sample diluent solution. Caspase-9 was measured by Caspase-9 Human ELISA Kit. The absorbance was taken at 450 nm. The data represent the mean of three independent experiments±SD. Statistical analysis were performed by t-test *, *p* < 0.05, compared with controls

A significant increase (*P* < 0.001) in the concentration of caspase-9 was observed through ELISA analysis of HL-60 cells treated with CB for 24 h as shown in Fig. 3. However, caspase-8 was not detected.

### Effect of CB on mitochondrial outer membrane permeability (MOMP)

Activation of caspase-9 is mostly depending on the release different proteins from the inner membrane space to the cytosol. Liberation of these proteins requires mitochondrial outer membrane permeabilization (MOMP).

**Fig. 4:**
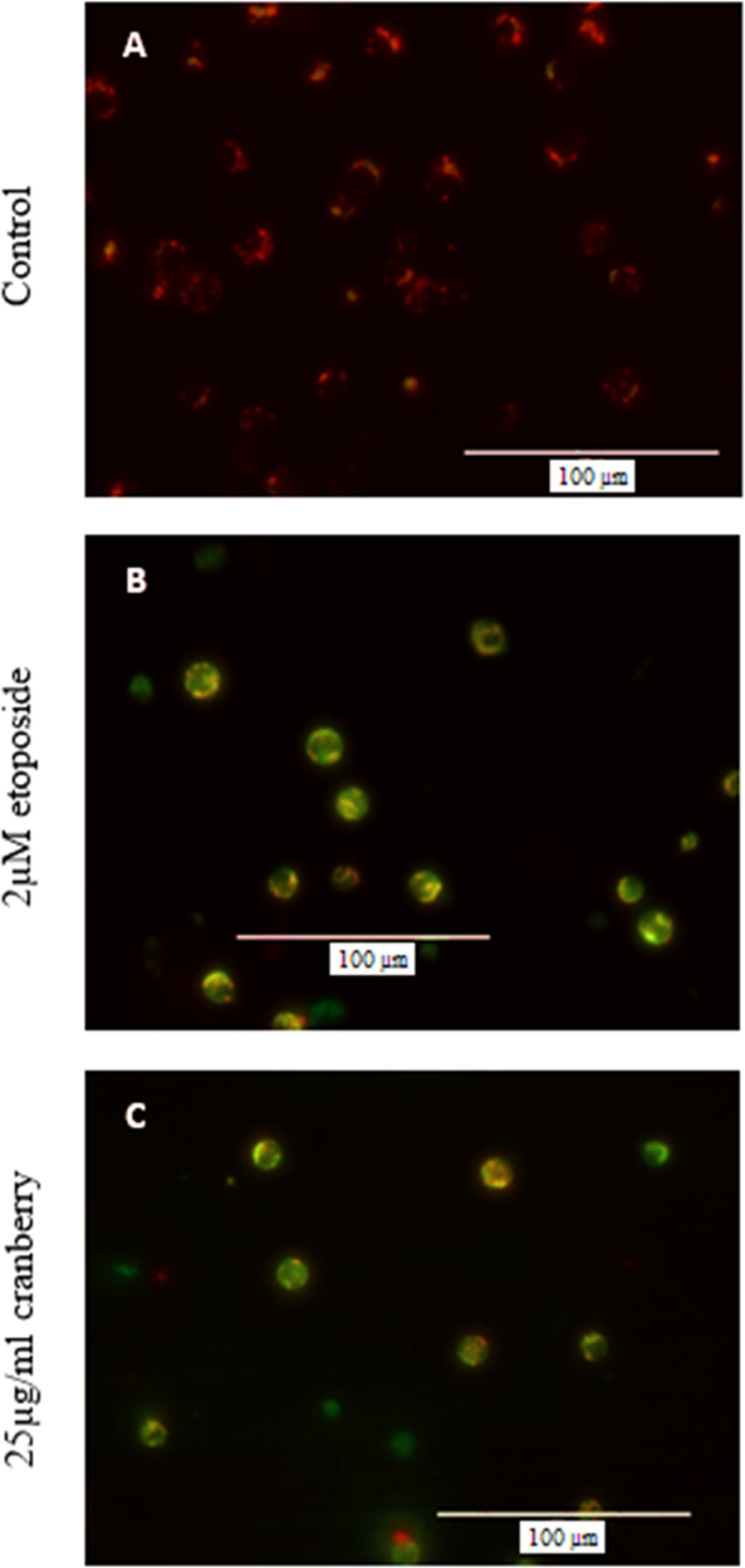
Effect of CB on Mitochondrial Transmembrane Potential in HL-60 cells. Cells were treated with 25 μg/mL CB for 24 h. Control or DMSO (0.1%)-treated cells served as controls. 2 μM etoposide used as positive control. Cells were dyed with MitoCapture dye. Fluorescent signals detected by fluorescence microscopy

24 h-CB-treated HL-60 cells showed stronger internal green fluorescence indicating an increase in MOMP, when compared to untreated cells. In addition, the fluorescence in cells treated with CB appeared as intense as observed in cells treated with the positive etoposide control (Fig. 4).

### Effect of CB on cytochrome C and Smac levels

**Fig. 5:**
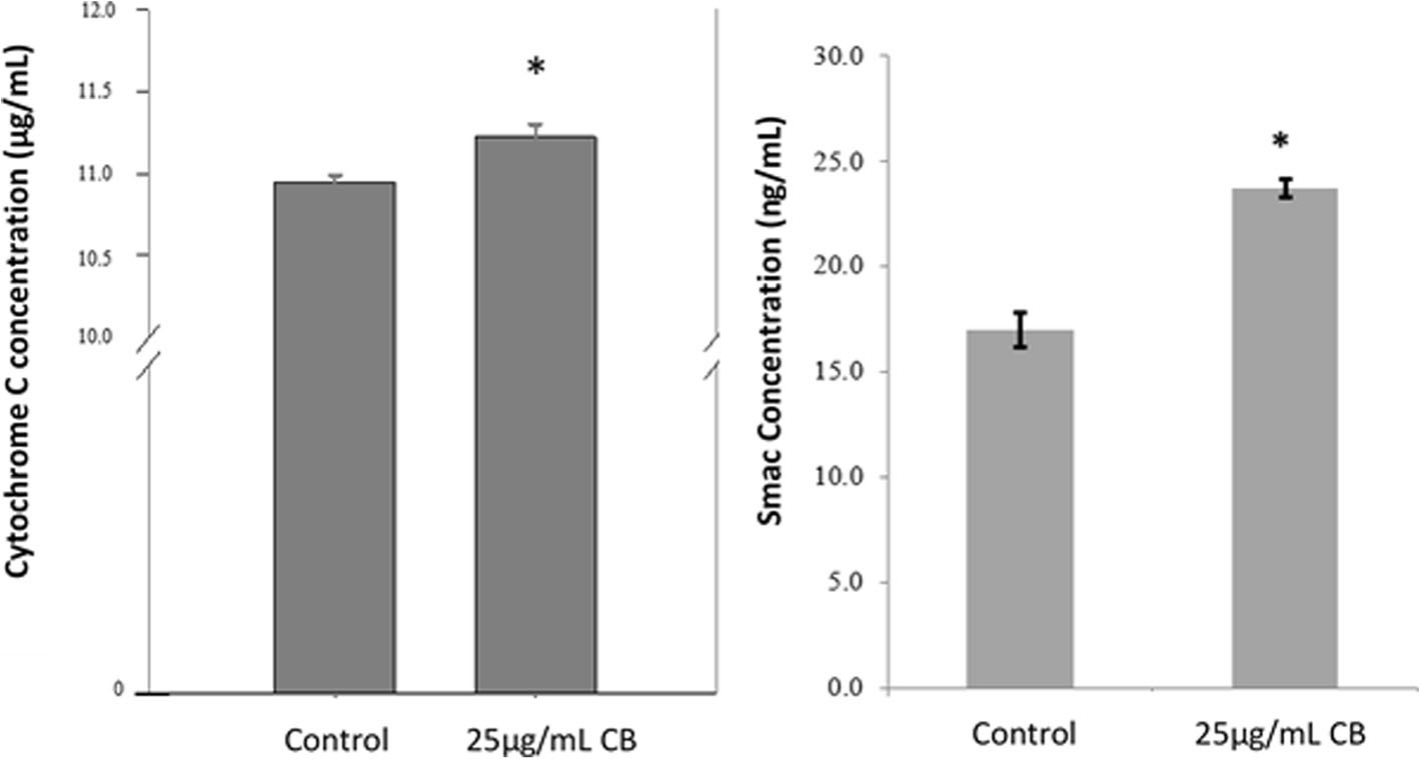
Change in cytochrome C and Smac concentrations. Cells were treated with 25 μg/mL CB for 24 h. Control or DMSO (0.1%)-treated cells served as controls. The data represent the mean of three independent experiments±SD. Statistical analysis were performed by t- test. *, *p* < 0.05

Cytochrome C and Smac are one of the major proteins that will be found in the cytosol after MOMP stimulation. In this study, ELISA results detected rises in their cytosolic concentrations in HL-60 cells that treated with CB for 24 h. Although the increasement detected was small in comparison to the untreated cells, the values were significantly (*P* < 0.001) different (Fig. 5). Together, the results came to support the previous data that the change in MOMP led to the release of the pro-apoptotic proteins; cytochrome C and Smac, from the inner membrane space of the mitochondria into the cytosol where they stimulate a cascade of caspase activation events leading to apoptosis.

### Effect of CB on the expression of BCL-2 family proteins

BCL-2 family proteins are known to play a major role in regulating MOMP and inhibiting/triggering intrinsic apoptotic pathways. The proteins subject to many post-translational modifications, specifically, phosphorylation. Therefore, total and phosphorylated form of some of BCL-2 family proteins were assessed.

#### Anti-apoptotic proteins

**Fig. 6:**
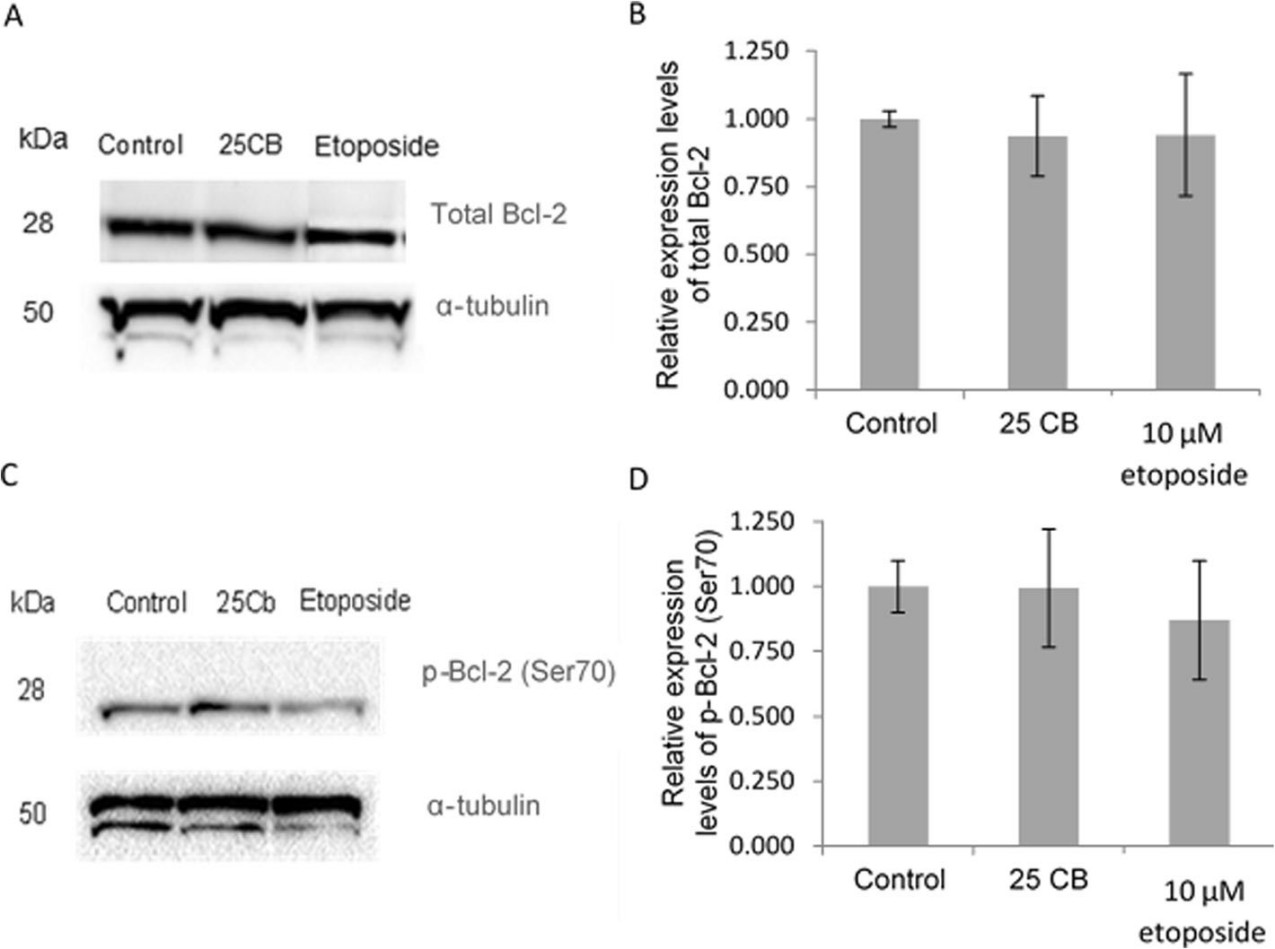
Expression of the anti-apoptotic BCL-2 protein in HL-60 cells treated with CB. Representative immunoblots and relative expression of (**a** + **b**) total BCL-2 and (**c** + **d**) p-BCL-2 (S70). Cells were treated for 24 h with 25 μg/mL CB, 10 μM etoposide or 0.1% DMSO. Total BCL-2 and p-BCL-2(S70) expression were normalized to α-tubulin. Values are mean ± SD, *n* = 3

Western blot detected no significant changes in cell lysates of HL-60 cells treated with CB for 24 h concerning relative protein expression levels of either total BCL-2 or its active form p-BCL-2 (Ser70) (Fig. 6).

#### Pro-apoptotic proteins

Since our results showed no change in BCl2 protein levels (both total and phosphorylated forms) we meant to focus on the role of the pro-apoptotic protein BAD as it can bind to the BCL2 and help in releasing the pro-apoptotic proteins Bax and Bak, those can trigger apoptosis .

**Fig. 7:**
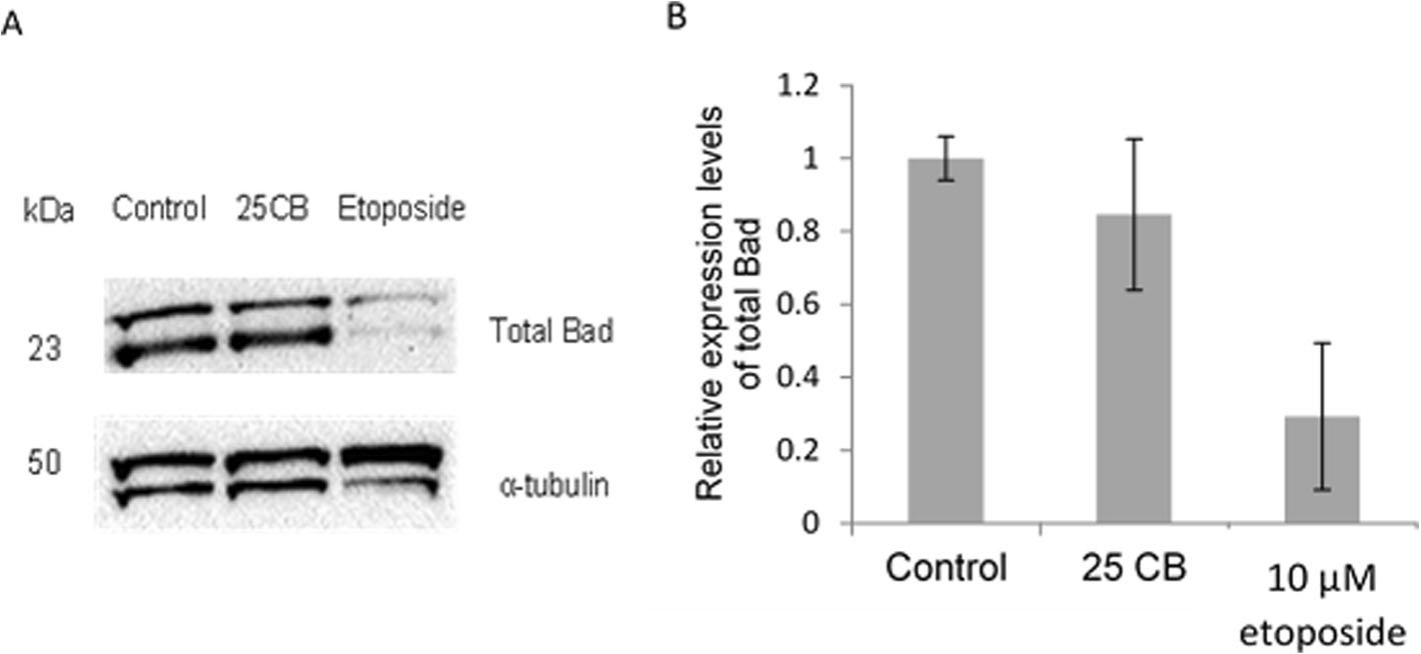
Expression of the pro-apoptotic BAD protein in HL-60 cells after treatment with CB. **a** Representative immunoblots and **b** relative expression of total BAD. HL-60 cells were treated with 25 μg/mL CB, 10 μM etoposide or 0.1% DMSO for 24 h. Total BAD expression was normalized to α-tubulin. Values are mean ± SD, n = 3

**Fig. 8:**
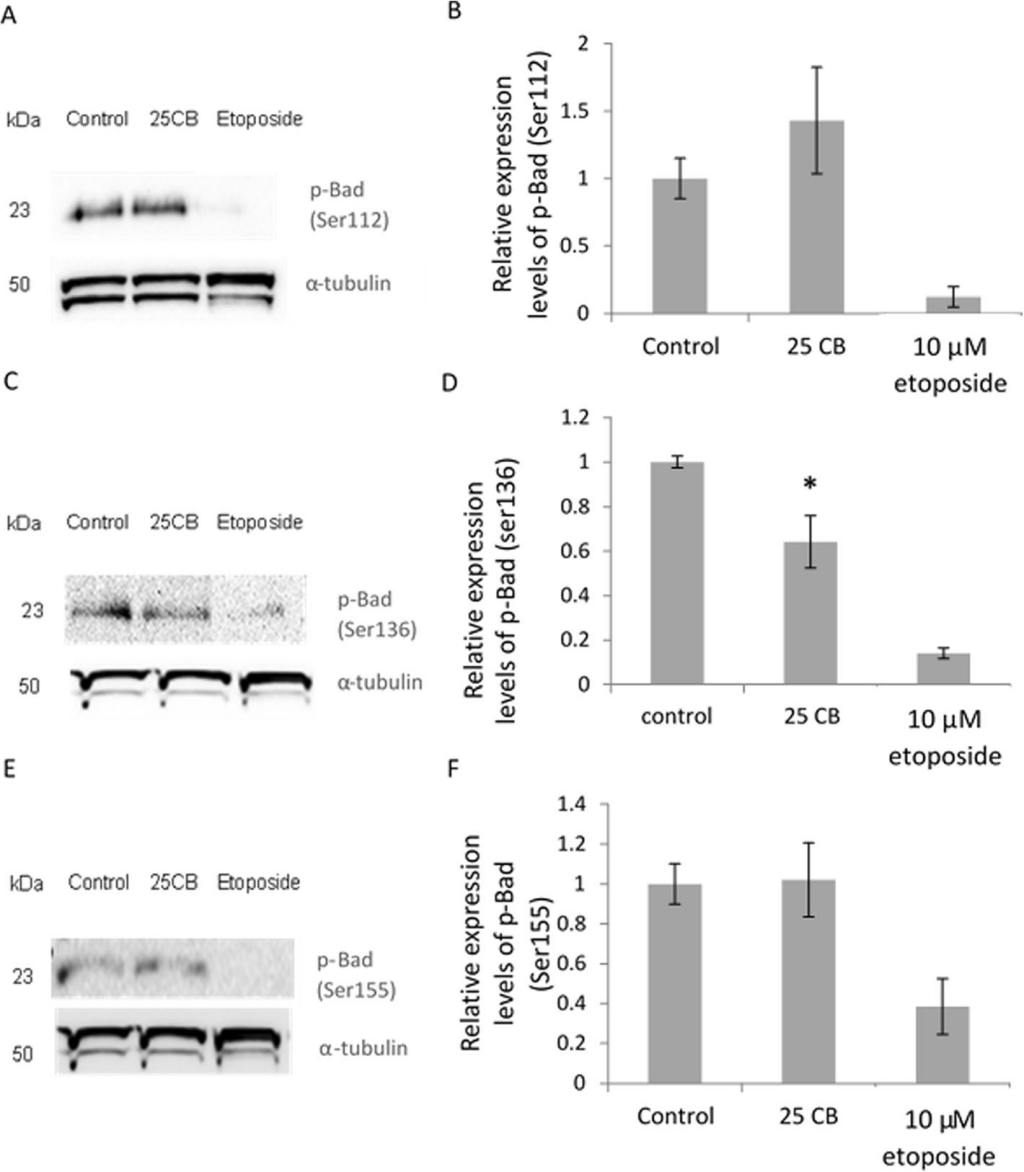
Expression of p-Bad in HL-60 under the effect of CB treatment. **a** Representative immunoblots of p-Bad (ser112) and α-tubulin. **b** Relative expression level of p-Bad (ser112). **c** Representative immunoblots of p-Bad (ser136) α-tubulin. **d** Relative expression level of p-Bad (ser136). **e** Representative immunoblots p-Bad (ser155) α-tubulin. **f** Relative expression level of p-Bad (ser155). HL-60 Cells were treated with 0.1% DMSO (control), 25 μg/mL CBor 10 μM etoposide (positive control) for 24 h. Expressions of p-Bad were normalized to α-tubulin expression as an internal control. Data represent mean ± SD from three independent experiments Statistical analysis were performed by one-way variance analysis (ANOVA) followed by Bonferroni’s test. Statistical difference shown as *P* < 0.05 [*]

Western blot analysis of cell lysates from 24 h- CB-treated HL-60 cells found that the treatment did not affect levels of total BAD (Fig. 7), or its phosphorylated (inactive) forms at Ser112 (Fig. 8a,b) and ser-155 (Fig. 8e,f). However, CB treatment did have a significant impact (*P* = 0.022) on the dephosphorylation of BAD at Ser136.

### Effect of CB on AKT phosphorylation

We investigated the involvement of Akt, which has been described as a downstream regulator of Bad.

**Fig. 9:**
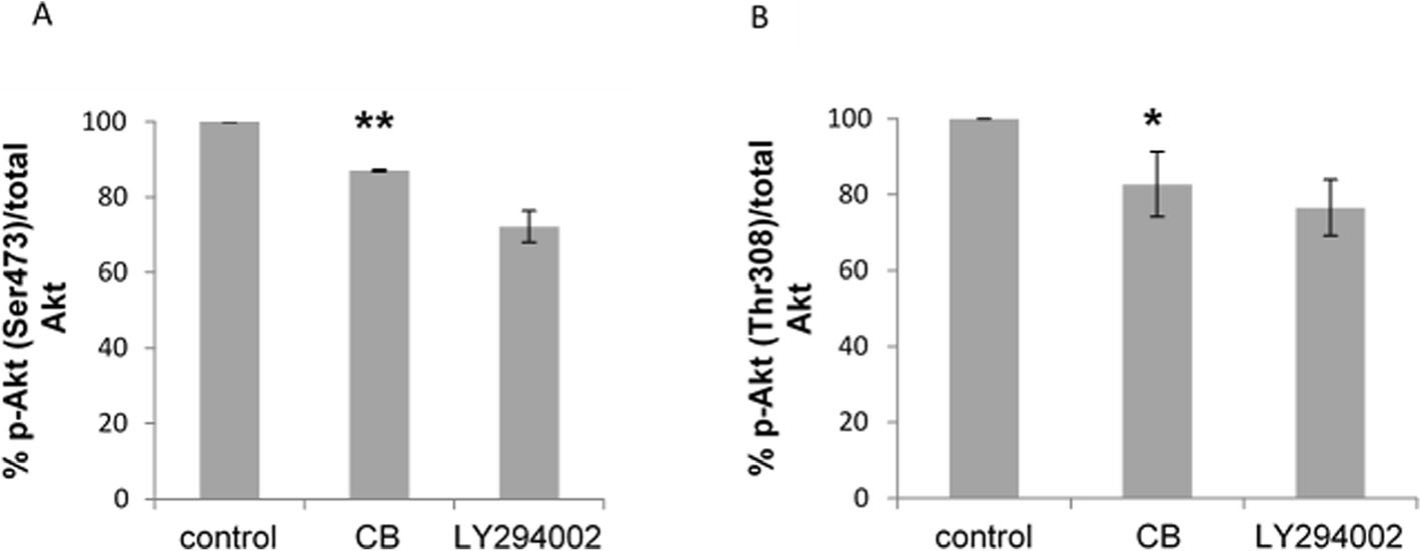
Inhibition of Akt phosphorylation in CB- treated HL-60 cell. Cells were treated with 0.1% DMSO (control), 25 μg/mL CB or 20 μM PI3K inhibitor LY294002 (positive control) for 24 h. cell lysates were assessed by ELISA for (**a**) p-Akt (Ser473) and (**b**) p-Akt (Thr308). Results are expressed as the percentage of p-Akt after normalized for total Akt. Values are the mean ± SD of 3 independent experiments. Statistical analysis were performed by one-way variance analysis (ANOVA) followed by Bonferroni’s test (p < 0.05). Statistical difference shown as *P* < 0.05 [*] and *P* < 0.001 [**]

ELISA analysis of HL-60 cells treated with CB for 24 h indicated a marked reduction of AKT phosphorylation at both Ser473 (*P* = 0.001) and Thr308 (*P* = 0.036) (Fig. 9a and b). This data revealed that 25 μg/mL cranberry treatment for 24 h can activate the pro-apoptotic Bad by inhibiting AKT phosphorylation at both Ser473 and Thr308 in HL-60.

## Discussion

The present study has defined the mechanism by which CB triggers apoptosis in HL-60 cells that provided an important molecular framework for the apoptosis initiated by CB. The lowest concentration of CB that initiates apoptosis has been determined. The preliminary results of the present study have showed that 24 h treatment of HL-60 cells with 25 μg/ml of CB activated two executioner caspases (3 and 7), and triggered apoptosis in those cells. The present study results are in agreement with few of the previous studies that 25 μg/ml of CB is effective in triggering apoptosis [18–20]. Cranberry juice contains PACs at a concentration of 418.8 ± 75.3 μg/ml; therefore, 25 μg/ml used in this study is appropriate within the PAC concentration of the juice itself [22].

In the intrinsic apoptotic pathway, caspase activation is associated with the permeability of the mitochondrial outer membrane (MOM) [23]. These results were similar to the present study stating that incubation of HL-60 cells with CB led to an increase in MOMP (Fig. 2). Few of the previous studies have also reported similar results stating that there is a significant impact of CB on MOMP [19, 24]. Caspase-mediated apoptosis can be induced through two major upstream pathways; intrinsic or mitochondrial, involving caspase-9 activation, or extrinsic or death receptor-related, which involves caspase-8 activation [3, 21]. The pathway used by apoptotic HL-60 cells after 24 h treatment with CB was determined by comparing the levels of caspases-8 and -9 in treated and untreated cells. The results of present study have shown that treatment with CB significantly increased the level of caspase-9; however, caspase-8 was not detectable in any of the samples.

Normally sequestered pro-apoptotic proteins, including cytochrome c and Smac, are released from the inter-membrane space into the cytosol when the MOM becomes permeable, which triggers a caspase-dependent mitochondrial pathway [3, 14]. MacLean et al. [18] study reported significant induction in the cytosolic- cytochrome C level in human DU145 prostate cancer cells under the effect of the whole cranberry extract treatment. Moreover, a recent study conducted by Kresty et al. [25] showed that cranberry treatment led to a significant increase in levels of cytosolic cytochrome C. On the other hand, no report was found to evaluate the effectiveness of PAC rich cranberry extract in triggering the release of Smac from the mitochondrial intermembrane space into the cytosol. However, the present study demonstrated that both cytochrome C and Smac were found in the cytosol of HL-60 cells treated with CB.

The balance between the anti-apoptotic and pro-apoptotic members of the BCL-2 family of proteins could be associated with an increase in MOMP. Moreover, the release of mitochondrial proteins is responsible for activating the caspase cascade of the intrinsic apoptotic pathway in the cytosol. BCL-2 family proteins are subjected to many post-translational modifications, specifically, phosphorylation. The functions of both BCL-2 and BAD proteins can be regulated by phosphorylation. The effect of cranberry on the level of expression of BCL-2 family proteins was evaluated by MacLean et al. [18], which showed that cranberry treatment caused an increase in BAX and tBID protein levels, but had no effect on the expression of the BCL-2 protein. While, phosphorylation of BCL-2 at a single site (Ser70) is needed for maintaining its function in an anti-apoptotic manner [26].

In the present study, HL-60 cells were treated with CB and looked for changes in the phosphorylation status of BAD, a pro-apoptotic protein, and BCL-2, an anti-apoptotic protein. The results showed a significant decrease in BAD phosphorylation at Ser136. However, none of the previous studies have evaluated whether CB interferes with post-translational phosphorylation of proteins within the BCL-2 family [19, 27, 28].

The present study has also investigated the involvement of AKT, a downstream regulator of BAD to elucidate the mechanism by which CB induced BAD activation. Previous literature has a model, in which active AKT phosphorylates BAD at Ser136 results in the dissociation of the BAD protein from BCL-2 [29, 30]. The protein is translocated into the cytosol resulting in subsequent binding/sequestation by 14–3-3 proteins leading to the inhibition of apoptosis and cell survival.

It has been hypothesized that CB was interfering with BAD phosphorylation by inhibiting AKT activity as the results indicated that CB reduced the phosphorylation of BAD at Ser136. Kvansakul & Hinds [31] treated SKOV-3 ovarian cancer cells with 50 μg/ml cranberry-PAC extract and demonstrated that apoptosis was induced through the activation of both intrinsic and extrinsic pathways. The results showed a clear reduction of AKT activation and a significant reduction of both p-AKT (Ser473) and p-AKT (Thr308) levels demonstrating association between extrinsic and intrinsic pathways at this concentration. Similar results were found in the study conducted by Singh et al. [19] using neuroblastoma cell lines.

The present study has provided experimental results to define a cascade of events to explain how might CB interferes with specific proteins in HL-60 cells and triggers apoptosis via the intrinsic apoptotic pathway. Treatment with CB in these cells inhibited AKT activation resulting in BAD activation. Active BAD led to increase in the permeability of the MOM, which caused the release of the pro-apoptotic protein’s cytochrome C and Smac into the cytosol. Presence of cytochrome C in the cytosol will activate caspase-9. Consequently, the executioner caspase-3 will be activated. Cytosolic-Smac, on the other hand, will facilitate caspase activation through neutralizing the endogenous IAPs.

However, one limitation of the present study is the use of a single cell line (HL-60). Therefore, applying different cell lines in future research could contribute to validate the findings of the current study. In addition, assessing the effect of CB treatment on normal cells in comparison to cancerous cells is also needed.

## Conclusion

The present study has explained the mechanism initiated by cranberry that triggers apoptosis in HL-60 cells. The results demonstrated that cranberry inhibited AKT activity in HL-60 cells, accompanied by a significant reduction of p-AKT (Ser473) and p-AKT (Thr308). The present study has suggested that link between cranberry-induced AKT inhibition and BAD activation, which results in the initiation of intrinsic apoptosis in HL-60 cells. It has provided novel insight into the sequence of events to describe the mechanism by which intrinsic apoptosis is triggered by CB in HL-60 cells.

## Data Availability

The datasets used and analyzed during the current study are available from the corresponding author on reasonable request.

## Abbreviations

AKT: Protein Kinase B
BAD: BCL-2-associated death promoter
BAX: BCL2-Associated X Protein
BCL-2: B-cell lymphoma-2
CB: Cranberry Extract
CO_2_: Carbon Dioxide
DMSO: Dimethyl Sulfoxide
ELISA: Enzyme-Linked Immunosorbent Assay
HRP: Horseradish Peroxidase
MOM: Mitochondrial Outer Membrane
MOMP: Mitochondrial Outer Membrane Permeability
PAC: Proanthocyanidins
PBS: Phosphate Buffered Saline
RIPA buffer: Radioimmunoprecipitation Assay Buffer
SDS-PAGE: Sodium Dodecyl Sulfate–Polyacrylamide Gel Electrophoresis
SMAC: Second Mitochondria-Derived Activator of Caspases
TBID: Truncated BH3 Interacting Domain Death Agonist
TBST: Tris Buffered Saline with Tween
